# DDSurfer: A Weakly‐Supervised Dual‐Stream Deep Learning Framework for Cortical Surface Reconstruction From Diffusion MRI

**DOI:** 10.1002/advs.76596

**Published:** 2026-07-23

**Authors:** Chengjin Li, Wei Zhang, Xi Zhu, Yuqian Chen, Nir A. Sochen, Jarrett Rushmore, Carl‐Fredrik Westin, Yogesh Rathi, Lauren J. O'Donnell, Ofer Pasternak, Fan Zhang

**Affiliations:** ^1^ University of Electronic Science and Technology of China Chengdu China; ^2^ Brigham and Women's Hospital Harvard Medical School Boston Massachusetts USA; ^3^ School of Mathematical Sciences University of Tel Aviv Tel Aviv Israel; ^4^ Department of Anatomy and Neurobiology Boston University School of Medicine Boston Massachusetts USA

**Keywords:** cortical surface reconstruction, deep learning, diffusion MRI, neuroimaging, weakly supervised learning

## Abstract

Cortical surface reconstruction of white matter and pial surfaces from diffusion MRI (dMRI) is critical for neuroimaging analyses, including tractography, connectomics, and multimodal data integration. However, obtaining these surfaces from dMRI data is inherently challenged by its low spatial resolution and poor tissue contrast. Currently, this relies on T1‐weighted images, from which the surfaces are reconstructed and then registered to the dMRI space—a process affected by inaccurate inter‐modality registration. This study introduces *DDSurfer*, an end‐to‐end deep learning framework that directly generates high‐fidelity cortical surfaces from dMRI data. DDSurfer leverages a novel dual‐stream architecture that processes and synergistically fuses complementary microstructural features from dMRI, learning a diffeomorphic transformation for subject‐specific surface reconstruction. The model is trained using a robust weakly‐supervised strategy with automatically generated pseudo‐ground‐truth surfaces. Extensive evaluations on diverse datasets demonstrate that DDSurfer surpasses traditional methods in geometric accuracy, morphological consistency, and generalization. By providing a computationally efficient and robust T1‐weighted‐independent solution, DDSurfer overcomes a major bottleneck in dMRI, delivering a practical tool to advance accurate dMRI‐centric connectomics and surface‐based investigations. Source code and implementation as an interactive 3D Slicer module are publicly available at: https://github.com/ChengjinLii/DDSurfer.

## Graphical abstract

DDSurfer reconstructs cortical surfaces directly from diffusion MRI without requiring T1‐weighted scans. By fusing complementary microstructural features and learning diffeomorphic deformations, it efficiently generates accurate white matter and pial surfaces, improving geometric fidelity and morphometric reliability across datasets for robust surface‐based connectomic and neuroimaging analysis.

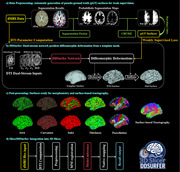

## 1 Introduction

Diffusion MRI (dMRI) is an advanced imaging technique that enables the quantification of tissue microstructural features [1] and in vivo mapping of white matter (WM) fiber tracts [2] through fiber tractography. In dMRI, cortical surface reconstruction of the inner WM and the outer pial surfaces, i.e., the two geometric boundaries that define the cortical ribbon, is a crucial step. This is essential in many computational and neuroscientific applications, enabling methodologies such as surface‐guided tractography, where the WM surface acts as an anatomical constraint for seeding streamlines [3, 4, 5], and surface‐guided region‐specific microstructure estimation, which uses surfaces to delineate regions for quantitative analysis [6, 7]. Furthermore, the reconstructed surfaces are important for investigating the structural relationship between the WM and cortex [8, 9], for example by facilitating the construction of brain structural connectomes [9]. However, the acquisition protocols of dMRI data, designed specifically to capture water molecule motion in brain tissues, inevitably lead to local distortions, noise artifacts, and reduced spatial resolution. As a result, accurate and efficient cortical surface reconstruction in dMRI continues to be a challenging task to resolve.

**FIGURE 1:**
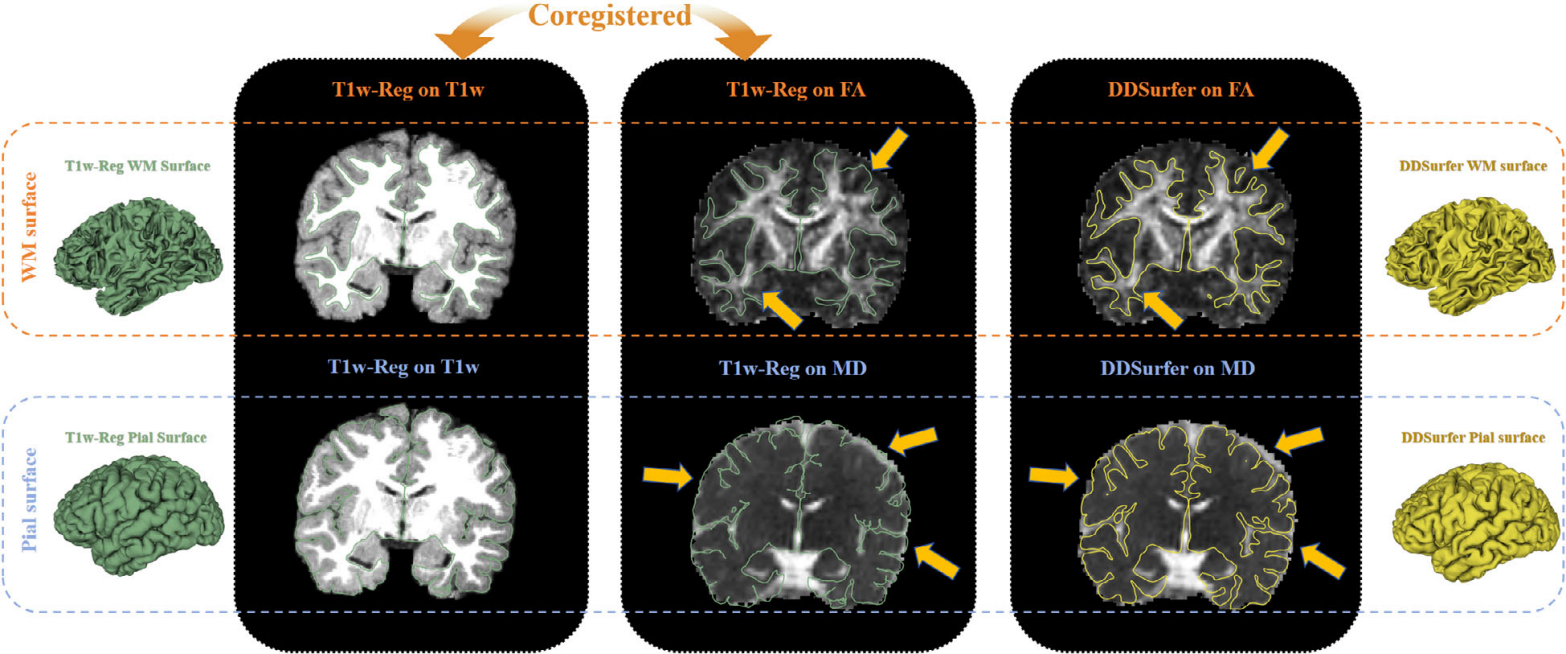
Comparison of cortical surface reconstruction in dMRI space. The green contour shows the surfaces that are reconstructed on the T1w image using FreeSurfer and then registered onto the dMRI image. This contour exhibits misalignment with the underlying cortical boundaries appearing on the FA and MD images. In contrast, the yellow contour shows our DDSurfer surfaces, generated directly from dMRI, which accurately delineate the cortical boundaries.

Currently, the widely used approaches for cortical surface reconstruction in dMRI rely on anatomical T1‐weighted (T1w) data, which provide high resolution and contrast required for surface reconstruction. Typically, the T1w image is first segmented into cortex, WM, and cerebrospinal fluid (CSF), from which boundaries between these tissues are extracted to generate an isosurface mesh (e.g., via marching cubes [10]) and subsequently corrected for topological errors such as holes (e.g., the fast marching algorithm [11]). In this way, the surfaces are computed and represented as continuous triangular meshes composed of vertices (points in 3D space) and faces (triangles connecting three vertices). Then, a nonlinear registration between the volumetric T1w and dMRI data is computed to warp the 3D surfaces to the dMRI space using the derived volumetric transforms. However, these approaches are time‐consuming and highly dependent on the quality of inter‐modality registration. This registration is often compromised by echo planar imaging (EPI) distortions [12, 13, 14] and the lower resolution of dMRI data [15], leading to potential misalignments between the registered surfaces and the underlying anatomical structures, as illustrated in Figure 1. Furthermore, these approaches are not applicable when anatomical T1w data are unavailable for various technical or procedural reasons. Therefore, there is a growing demand for cortical surface reconstruction methods that can operate directly on dMRI data.

Advances in computational dMRI have shown that tasks such as tissue segmentation and brain parcellation can be performed effectively using dMRI directly [16, 17, 18, 19, 20], rather than using anatomical T1w data. One emerging strategy is to synthesize structural MRI contrasts [21, 22, 23], such as T1‐weighted images, from dMRI and then apply established cortical surface reconstruction tools such as FreeSurfer [24]. Another category of methods directly predicts segmentation on dMRI without inter‐modality synthesis. For example, recently proposed approaches have demonstrated successful coarse‐scale tissue segmentation into the entire WM, gray matter (GM), and cerebrospinal fluid (CSF) [20] and fine‐scale cortical parcellation into over 100 subregions from dMRI data [18, 19]. These studies provide compelling support for the feasibility of cortical surface reconstruction directly from dMRI data, as the process fundamentally relies on accurately defining the boundaries between different tissue types. A common feature of these methods is leveraging the unique multi‐parameter characteristics of dMRI, where each parameter provides distinct structural insights for delineating specific brain structures. For instance, fractional anisotropy (FA) quantifies directional variation in water diffusion and proves highly discriminative in describing WM regions characterized by high diffusion anisotropy, while mean diffusivity (MD) measures the overall magnitude of water diffusion and shows greater discriminative power for cerebrospinal fluid (CSF) regions exhibiting free water movement [25]. Although the post‐processing pipelines designed for T1w data can be adapted for the resulting dMRI segmentations for surface reconstruction, the accuracy is often compromised by the low spatial resolution and partial volume effects inherent to dMRI, as well as by any imprecision in the initial tissue segmentation.

There has been extensive work on cortical surface reconstruction in neuroimaging. Existing methods have primarily focused on anatomical T1w data [26, 27], with recent deep learning advances demonstrating particularly successful fast and accurate reconstruction methods [28, 29, 30, 31, 32, 33, 34, 35, 36, 37, 38, 39, 40]. While these methods provide a technical foundation for dMRI‐based surface reconstruction, directly applying T1w‐optimized approaches to dMRI remains challenging due to fundamental differences in their contrast mechanisms and resolution characteristics. In general, existing T1w‐based deep learning methods can be divided into two categories, i.e., *implicit* and *explicit*. The implicit methods [35, 36, 37, 38, 40] predict intermediate voxel‐wise surface representations (e.g., signed distance function, SDF), followed by post‐processing methods for mesh extraction and topology correction. While the implicit methods [35, 36, 37, 38, 40] can improve efficiency compared to traditional methods (e.g., FreeSurfer [24]), their performance is highly affected by partial volume effects, as low‐resolution dMRI often contains mixed tissues within boundary voxels, which is a particularly prevalent challenge in the low‐resolution dMRI data. On the other hand, the explicit methods directly generate 3D surfaces by optimizing mesh deformation [28, 29, 30, 31, 32, 33, 34] by learning a deformation to warp an initial mesh surface (usually a groupwise surface template) into the target space for surface reconstruction. However, the majority of existing explicit methods are supervised learning approaches that rely on large‐scale, high‐quality ground‐truth surfaces generated using traditional methods (e.g., FreeSurfer) for model training [28, 29, 30, 31, 32, 33, 34]. As a result, such methods cannot be directly applied to dMRI data as there have been no automated methods that can generate ground‐truth surfaces (corresponding to FreeSurfer results on T1w data) in large‐scale dMRI data yet. The recently proposed CoSeg method [39] provides a novel weakly‐supervised learning method that uses voxel‐level segmentation results as pseudo‐ground‐truth (pGT), without requiring precise ground‐truth surfaces. The overall idea is to learn diffeomorphic surface deformations that warp an initial topologically valid template surface onto a target cortical surface space [29, 31, 33, 39]. The process is guided by a weakly‐supervised loss function, which includes a surface spatial alignment term that ensures the warped surface generally aligns with the pGT boundary. Additional regularization terms are incorporated such as an inflation term to keep the deformation adhering to its normal direction [34, 39], an edge length loss to prevent abrupt normal flips between adjacent faces [28, 41], and normal consistency terms to discourage irregular edge stretching and jagged local geometry [28, 41]. Together, these losses guard against pseudo‐label errors to prevent inaccuracies in the pGT data from being propagated. This method suggests a promising hybrid implicit‐to‐explicit strategy to address the challenges of low resolution and the absence of ground‐truth surfaces for dMRI mesh surface reconstruction.

In light of the above, this paper proposes *DDSurfer*, a novel end‐to‐end deep learning framework for cortical surface reconstruction directly from dMRI. Our paper has the following major contributions. First, DDSurfer enables automated, single‐stage reconstruction of WM and pial surfaces from dMRI without the need for anatomical T1w data. Second, we introduce a weakly‐supervised deep learning framework that trains on voxel‐level segmentation as pGT surfaces, eliminating the need for precise surface‐level annotations in dMRI data. Third, we design a novel network architecture that includes: (1) a dual‐stream input processing module to leverage complementary information from dMRI‐derived anisotropy and diffusivity parameter maps, and (2) a hybrid surface prediction module to compute implicit surface representations and subsequently explicit geometric 3D mesh surfaces. Fourth, DDSurfer can seamlessly integrate with FreeSurfer post‐processing steps for further surface analyses, such as surface parcellation and thickness estimation. Fifth, we demonstrate DDSurfer's good generalization capability on a large test dataset from 5 independently acquired populations across different ages and multiple health conditions. The code for DDSurfer is released publicly at https://github.com/ChengjinLii/DDSurfer. Finally, a 3D Slicer plugin is developed to facilitate direct execution and visualization of DDSurfer within the popularly used open‐source 3D Slicer platform.

## 2 Methods

**FIGURE 2:**
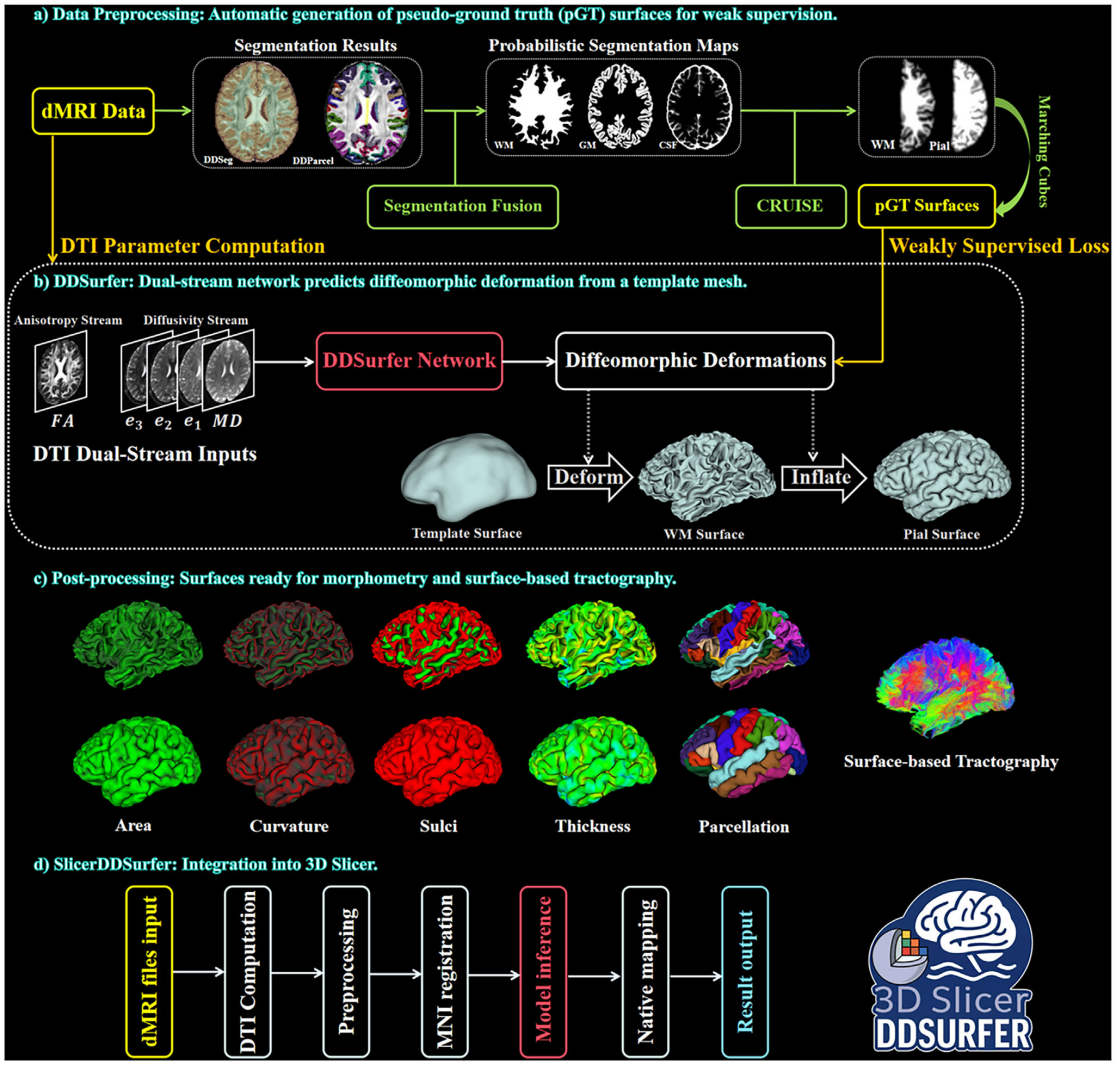
Graphic overview of the DDSurfer Framework. (a) Data preprocessing to generate pGT WM and pial surfaces from dMRI data; (b) DDSurfer network model to predict the surfaces from dMRI data; (c) Post‐processing to enable downstream surface‐based tasks; (d) SlicerDDSurfer software to integrate the proposed DDSurfer method into the 3D Slicer platform for interactive, interface‐based usage.

DDSurfer is an end‐to‐end, dual‐stream deep learning framework for direct dMRI‐based cortical surface reconstruction (see Figure 2 for an overview). The methodology comprises four main stages: (a) an automated pipeline for generating pGT WM and pial surfaces for weakly supervised learning, (b) the DDSurfer model that learns diffeomorphic deformations to map a template surface mesh for subject‐specific surface reconstruction, (c) a post‐processing workflow for morphometric analysis and surface‐based tractography, and (d) DDSurfer is integrated into the 3D Slicer platform, providing an accessible toolkit for analyzing cortical surfaces using dMRI data.

### 2.1 Pseudo‐Ground‐Truth (pGT) Surface Generation

We design an automated strategy to generate pGT surfaces for weakly supervised learning. Unlike T1w data, for which established tools like FreeSurfer can generate high‐fidelity cortical surfaces used as ground‐truth when developing alternative methods, no analogous method exists for producing ground‐truth surfaces from dMRI data. To address this, we develop a dMRI‐specific pipeline to generate implicit surface representations and subsequently reconstruct rough WM and pial surfaces.

We first compute the brain segmentations using two deep learning models specifically designed for dMRI data: *DDSeg* [20], which provides tissue‐level WM/GM/CSF segmentations, and *DDParcel* [18], which provides parcel‐level cortical and subcortical segmentations based on Desikan–Killiany (DK) atlas parcellation [42]. A key advantage of both approaches is their ability to derive output segmentations directly from dMRI data. Moreover, as part of the results, they compute voxel‐wise probabilistic maps for each segment, which are required for subsequent signed distance function (SDF) computation. The two methods complement each other for the SDF computation, where DDSeg provides the overall tissue posteriors needed to define the WM–GM and GM–CSF interfaces, while DDParcel can be used to derive a cortical mask that excludes non‐cortical regions (e.g., ventricles and subcortical structures) from the tissue posteriors. For WM surface reconstruction, we compute the WM probability map from the masked WM/GM posteriors. For pial surface reconstruction, DDSeg captures the cortical boundary against CSF, which is essential for defining the outer surface, and we similarly compute the pial probability map from the masked GM/CSF posteriors.

Finally, the processed probability maps are converted into continuous SDFs for WM and pial surfaces using the Cortical Reconstruction using Implicit Surface Evolution (CRUISE) algorithm [43]. Specifically, the WM SDF is computed from the WM and GM probability maps, while the pial SDF is computed from the GM and CSF probability maps. The marching cubes method [10] is then applied to extract the corresponding surface meshes. These surfaces, sufficiently accurate for both tissue completeness and boundary definition, serve as pGT surfaces for subsequent DDSurfer model training. No additional topology correction is applied at the pGT generation step, because such correction is computationally expensive and can itself introduce new local geometric errors when applied to coarse dMRI‐derived SDFs.

### 2.2 DDSurfer Network

**FIGURE 3:**
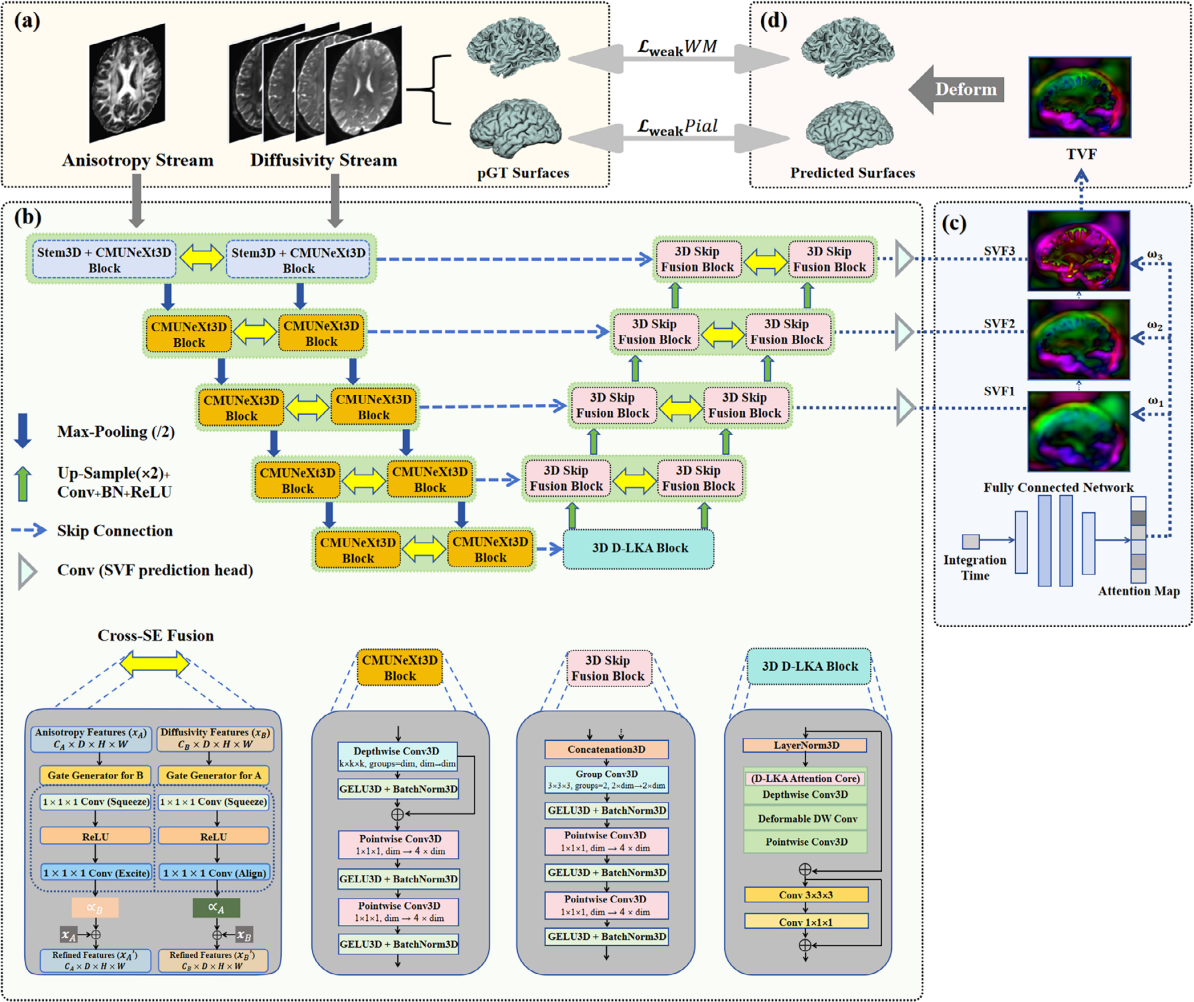
DDSurfer network architecture. (a) Dual‐stream network input derived from dMRI data; (b) DualStream–FuseNet for image feature computation; (c) DiffeoSurf–FlowNet for surface deformation estimation. (d) Weakly supervised learning using pGT surfaces.

Figure 3 gives an overview of the DDSurfer network workflow. First, the network processes a set of input dMRI parameter maps (Section 2.2.1) via a DualStream–FuseNet subnetwork to compute multi‐scale feature maps that encode the dMRI‐derived tissue microstructural information (Section 2.2.2). Then, diffeomorphic surface deformations are computed via a learnable DiffeoSurf–FlowNet subnetwork to warp a template surface into individual space, enabling subject‐specific reconstruction of the WM and pial surfaces (Section 2.2.3). Finally, leveraging the computed pGT surfaces for loss computation, the warped surfaces are optimized in a weakly‐supervised manner that simultaneously enforces geometric fidelity and mesh regularity (Section 2.2.4).

#### 2.2.1 Network Input

Effective surface reconstruction requires input data that is descriptive of tissue boundaries across different brain structures. In our work, we chose the diffusion tensor imaging (DTI) model [1] for its simplicity and compatibility with legacy data. Five widely used DTI‐derived parameter maps are adopted, including FA, MD and the three tensor eigenvalues, which have been shown effective for tissue segmentation in our previous work [18, 20]. These parameter maps are partitioned into two categories based on their biophysical relevance to specific tissue interfaces. The first category includes the FA map, referred to as the *Anisotropy* category, that provides high contrast at the gray/white matter boundary. The second category includes the MD and eigenvalue maps, referred to as the *Diffusivity* category, that provide high contrast at the pial surface against the CSF.

#### 2.2.2 Subnetwork: DualStream–FuseNet

To leverage the complementary information from the two input parameter categories, we propose a DualStream–FuseNet subnetwork for feature computation. Our key architectural contribution is a cross‐stream fusion mechanism that includes a dual‐stream U‐shape encoder‐decoder for coarse‐to‐fine feature extraction from each input stream, integrated with a feature fusion module to facilitate interaction between the two streams. Figure 3b gives the network diagram of DualStream–FuseNet. The underlying architecture is based on the recently proposed CMUNeXt [44], which is a lightweight network for global context feature extraction. We chose CMUNeXt for easier implementation and wider deployment (see Section 3.2), because of its high efficiency at reduced computation cost on standard machines. For each of the two input streams, the encoder path begins with a 3D stem block to extract initial features at input image resolution, followed by 4 CMUNeXt 3D blocks with depthwise separable convolution [45] to compute hierarchical features at downsampled resolutions. Then, the decoder path includes 4 Skip Fusion blocks with group convolutions [46] to integrate upsampled features with corresponding high‐resolution encoder features via skip connections. The output features from the decoder blocks are then integrated into the following DiffeoSurf–FlowNet (see Section 2.2.3) for deformation computation.

To enhance the interaction between the two streams, we design a Cross‐Stream Squeeze‐and‐Excitation (Cross‐SE) Fusion module to perform feature fusion at each level of encoders and decoders. This module extends the squeeze‐and‐excitation [47] concept into a cross‐stream gating form, enabling bidirectional recalibration rather than simple concatenation. In detail, for each encoder and decoder block, the Anisotropy stream $x_{\alpha }$ is processed through a lightweight excitation pathway to produce an adaptive gating map that modulates the Diffusivity stream $x_{\beta }$, generating refined features $x_{\beta }^{\prime }=x_{\beta }⊙\sigma \;\left(f(x_{\alpha })\right)$. Similarly, $x_{\beta }$ generates a corresponding gate to recalibrate $x_{\alpha }$, yielding $x_{\alpha }^{\prime }=x_{\alpha }⊙\sigma \;\left(f(x_{\beta })\right)$. Here, σ(·) denotes the element‐wise sigmoid that constrains the gate to [0,1]; f(·) denotes a lightweight SE excitation mapping implemented as two 1×1×1 pointwise convolutions with a ReLU between them; ⊙ denotes element‐wise multiplication with broadcasting over spatial dimensions; and $x_{\alpha }^{\prime }$/$x_{\beta }^{\prime }$ indicate the recalibrated features. After Cross‐SE fusion at the current level, the two streams continue in parallel to the next, where the input is now enriched with complementary information from the other stream. At the bottleneck level, we further include a shared 3D Deformable Large Kernel Attention (D‐LKA) Block [48] that is utilized to model long‐range spatial dependencies and integrate information from both streams.

#### 2.2.3 Subnetwork: DiffeoSurf–FlowNet

From the computed features, we propose a DiffeoSurf–FlowNet subnetwork to reconstruct the cortical surfaces. DiffeoSurf–FlowNet is based on CoSeg [39], which is a flow‐based diffeomorphic deformation model. In this model, a cortical surface is represented as the vertices on a 3D template mesh, where each vertex is mapped to the subject‐specific space through a learned deformation field formulated as a time‐varying velocity field (TVF) [49] as shown in Figure 3d. Extending CoSeg, we introduce a hierarchical network design to leverage the multi‐scale features from the above DualStream–FuseNet. This design can enable a coarse‐to‐fine deformation prediction by processing features from low to high resolution [49, 50].

Specifically, at each output feature scale from DiffeoSurf–FlowNet, a convolutional head is used to compute a set of stationary velocity fields (SVFs). The SVFs are integrated via a weighted sum scheme where the weights are computed from time‐varying attention maps predicted by a fully connected network. This yields the TVF at each scale, as shown in Figure 3c. Then, across the multiple scales, the TVF with lower resolution is upsampled and accumulated with the upper scale to obtain the final TVF, as shown in Figure 3d. Finally, we integrate this TVF over time using a fixed‐step fourth‐order Runge–Kutta scheme to obtain a diffeomorphic deformation field, which is then applied to all template vertices. To reconstruct the two surfaces of WM and pial, two sequential diffeomorphic deformations are predicted: warping a template surface to the individual WM surface, followed by inflating the resulting WM surface to reconstruct the pial surface.

For the WM surface, DiffeoSurf–FlowNet predicts a diffeomorphic deformation that warps a topologically valid WM template surface computed from the Montreal Neurological Institute (MNI) average template [39, 51] into each subject's dMRI space. The template vertices are advected from time 0 to 1 under the learned atlas‐to‐WM TVF, and their final positions directly define the reconstructed WM surface. For the pial surface, we treat the reconstructed WM surface as the template and learn a second, pial‐directed TVF that effects an inflation from the WM surface to the outer pial surface. Unlike the WM stage (template‐to‐WM deformation), this pial stage is encouraged by an inflation loss to move mainly along the WM outward normals (see Section 2.2.3). In practice, we reuse the same SVF dictionary and weighting head to generate this Pial‐directed TVF, so that the pial inflation is modeled as a residual deformation on top of the WM stage, reducing redundancy and promoting WM–pial geometric consistency.

#### 2.2.4 Loss Function Design

The derived WM and pial surfaces from DiffeoSurf–FlowNet are optimized against the pGT surfaces in a weakly‐supervised manner to handle the inherent imperfections in the pGT data. The models for the WM and pial surfaces are trained separately.

For the WM surface model, the training is guided by a loss function with a primary reconstruction term and two geometric regularization terms. The reconstruction term is the standard bidirectional Chamfer distance loss term ($L_{\mathrm{Chamfer}}$), which ensures geometric alignment between the predicted surface and the pGT WM surface. This is supplemented by a normal consistency loss term ($L_{\mathrm{nc}}$) to maintain mesh smoothness and an edge length loss term ($L_{\mathrm{edge}}$) to preserve edge uniformity [28, 41]. Here, $L_{\mathrm{nc}}$ is computed from pairwise face‐normal consistency over neighboring triangles, which penalizes abrupt local changes in surface orientation, and $L_{\mathrm{edge}}$ is computed from the variation of edge lengths across the mesh, which penalizes irregular local stretching and jagged geometric artifacts [28, 41]. Together with the diffeomorphic template‐warping formulation, this surface‐based loss design helps preserve a valid cortical topology, even though the pGT WM surface has the topological defects often introduced by marching‐cubes‐based isosurface extraction. The total loss for the WM surface is defined as:

(1) $$L_{\mathrm{WM}}=L_{\mathrm{Chamfer}}+w_{\mathrm{nc}}\;L_{\mathrm{nc}}+w_{\mathrm{edge}}\;L_{\mathrm{edge}}.$$

For the pial surface model, a two‐stage training strategy is employed to account for pGT inaccuracies in deep sulci. An initial pre‐training stage uses a mean squared error (MSE) loss to encourage an outward inflation from the predicted WM surface, providing a good initialization. The main training then switches to a loss term centered on a unidirectional boundary loss ($L_{\mathrm{boundary}}$). This term computes a one‐way Chamfer distance from the pGT surface to the predicted surface, so that each coarse pGT location must be covered while extra predicted vertices are not penalized for extending deeper into the sulcus beyond the pGT. Let $V_{\mathrm{Pial}}$ and ${\hat{V}}_{\mathrm{Pial}}$ denote the predicted and pGT pial vertices. We define

(2) $$L_{\mathrm{boundary}}\left(V_{\mathrm{Pial}},{\hat{V}}_{\mathrm{Pial}}\right)=\frac{1}{|{\hat{V}}_{\mathrm{Pial}}|}\sum_{y\in {\hat{V}}_{\mathrm{Pial}}}\min_{x\in V_{\mathrm{Pial}}}{∥x-y∥}_{2}^{2}.$$

Additionally, an inflation loss term $L_{\mathrm{inflation}}$ [39] encourages the pial vertices to expand roughly along the normal direction of the WM surface, thereby favoring outward motion over arbitrary tangential drift. In this way, the unidirectional boundary loss allows the surface to extend beyond underestimated pGT boundaries in deep sulci, while the inflation term keeps that extension anatomically plausible with respect to the underlying WM surface. Combined with the same normal consistency and edge uniformity terms, the total loss for the pial surface is:

(3) $$L_{\mathrm{Pial}}=L_{\mathrm{boundary}}+w_{\mathrm{inflate}}\;L_{\mathrm{inflation}}+w_{\mathrm{nc}}\;L_{\mathrm{nc}}+w_{\mathrm{edge}}\;L_{\mathrm{edge}}.$$

### 2.3 Post‐DDSurfer Analyses and Downstream Applications

The DDSurfer outputs of WM and pial surfaces are compatible with the standard FreeSurfer neuroimaging pipeline, enabling a range of cortical surface analyses such as cortical parcellation, surface curvature and area computation, and cortical thickness estimation (see Figure 2c). For cortical parcellation, after importing the DDSurfer‐generated WM and pial surfaces into the standard FreeSurfer format, we map each cortical surface to a spherical coordinate system and then nonlinearly align them to the population fsaverage template, thereby establishing vertex‐to‐vertex correspondence between the subject‐specific and the template surfaces. This allows us to directly map any existing parcellation schemes, such as the anatomical Desikan–Killiany (DKT) atlas [42] and the functional Yeo atlases [52], defined in the template space onto the individual subject surfaces. For morphological analysis, the same FreeSurfer‐compatible surfaces are further processed by a standard surface‐analysis pipeline: vertex‐wise cortical thickness is estimated as the distance between corresponding WM and pial vertices, surface area is computed from the local triangular tessellation, and curvature and sulcal depth are derived from inflated surfaces, all using off‐the‐shelf FreeSurfer utilities consistent with the conventional FreeSurfer workflow. The computed surfaces and derived metrics enable various downstream applications, such as quantitative cortical thickness analysis and surface‐based tractography. This makes the method a useful tool for providing high‐quality inputs to connectomics and morphometry research.

### 2.4 Implementation Details and SlicerDDSurfer

Our method is implemented using PyTorch (v1.7) [53]. Model training is performed on a Linux workstation equipped with NVIDIA RTX 3090 GPUs. Adam is used as the optimizer with a batch size of one. The initial learning rate is set to 0.0005 and is dynamically adjusted using a cosine annealing scheduler [54]. The composite loss function is governed by empirically set weights: the normal consistency loss weight $w_{\mathrm{nc}}$ is 2.5, and the edge length loss weight $w_{\mathrm{edge}}$ is 0.5. For the pial surface model, which undergoes a special two‐stage training strategy, the inflation loss weight $w_{\mathrm{inflate}}$ is set to 2.0. This pial model is first pre‐trained for 20 epochs with the MSE loss, with the target being a surface generated by inflating the WM surface by an inflation factor of 2. Following pre‐training, the model is fine‐tuned using the full weakly‐supervised loss. The network takes a five‐channel volume (composed of FA, MD, and three eigenvalues) and an initial 160k vertex template mesh [39] as input to predict the final surface deformation.

To enhance accessibility and facilitate practical use in neuroimaging research, we integrate DDSurfer into the 3D Slicer platform as an interactive module, SlicerDDSurfer (Figure 2d). The SlicerDDSurfer interface provides a unified graphical environment for diffusion‐derived cortical surface reconstruction, enabling users to perform preprocessing, model inference, and post‐processing without command‐line operations. As illustrated in Figure 2d, the workflow consists of several automated steps: (1) users specify input and output directories and subject ID, (2) diffusion MRI data are loaded and preprocessed, (3) rigid registration to MNI space is performed [51], (4) cortical surfaces are predicted by the trained DDSurfer network, (5) results are transformed back to native space, and (6) the reconstructed surfaces are exported for downstream analyses.

## 3 Experimental Section

We perform experiments on multiple independently acquired MRI datasets across different populations with varying ages and health conditions, and acquired using varying acquisition protocols and scanners (Section 3.1). Extensive experimental evaluations are performed, including: (1) state‐of‐the‐art comparison to benchmark performance and establish optimal accuracy (Section 3.2), (2) ablation studies to evaluate the contribution of each component in our network design (Section 3.3), (3) test–retest assessment to evaluate the method's reproducibility and reliability (Section 3.4), (4) direct geometric validation against expert‐annotated reference surfaces (Section 3.5), (5) computational efficiency to evaluate processing speed and resource requirements (Section 3.6), (6) generalization analysis to demonstrate robustness across the diverse datasets (Section 3.7), and (7) downstream application to showcase the method's practical utility in surface‐based tractography (Section 3.8).

### 3.1 Datasets and Preprocessing

Data from five independently acquired datasets are used, including: 1) the Human Connectome Project Young Adult (HCP‐YA) [55], 2) the Autism Brain Imaging Data Exchange (ABIDE II‐NYU) [56], 3) the Consortium for Neuropsychiatric Phenomics (CNP) [57], 4) the Parkinson's Progression Markers Initiative (PPMI) [58], and 5) our in‐house adolescent depression (IHAD) dataset. Among these datasets, the HCP‐YA data, acquired with an advanced acquisition protocol featuring a high spatial and angular resolution, are used for model training, validation and testing. The other datasets, acquired with protocols geared toward clinical applications, are used to demonstrate how DDSurfer generalizes to data from different sources.

#### 3.1.1 Demographics and Data Acquisition Protocols

The HCP‐YA dataset includes 500 young healthy adults (age: 29.1±3.7 years; 250 females, 250 males) randomly selected from the HCP database. The HCP‐YA data are acquired on a customized 3T Siemens Skyra scanner. The acquisition parameters used for the dMRI data are TE = 89.5 ms, TR = 5520 ms, and voxel size = $1.25\times 1.25\times 1.25\;{\mathrm{mm}}^{3}$. The accompanying T1w images are acquired with a voxel size = $0.7\times 0.7\times 0.7\;{\mathrm{mm}}^{3}$, TE = 2.14 ms, and TR = 2400 ms. In our study, we split the HCP‐YA dataset into training (60%), validation (10%), and testing (30%) sets for model development and evaluation. Additionally, we use the HCP‐YA test–retest dataset from another 44 subjects (age: 30.4±3.3 years; 31 females, 13 males) to assess the reliability of our method. The ABIDE II‐NYU dataset includes 52 subjects (age: 10.24±5.74 years; 27 females, 25 males), acquired on a 3T Siemens Allegra scanner, with acquisition parameters including a b‐value of $1000\;s/{\mathrm{mm}}^{2}$, 65 directions, and TR/TE = 5200/78 ms, with a resolution of 3mm isotropic. The CNP dataset includes randomly selected 50 young adults (age: 37.6±9.2 years; 20 females, 30 males) including healthy controls and patients with various neuropsychiatric disorders, with data acquired on a 3T Siemens TrioTim scanner at 2mm isotropic resolution, TR/TE = 9000/93 ms, and 64 gradient directions at $b=1000\;s/{\mathrm{mm}}^{2}$. The PPMI dataset includes randomly selected 50 elderly adults (age: 62.8±7.1 years; 25 females, 25 males) including healthy controls and Parkinson's disease patients, with data acquired on a 3T Siemens TrioTim scanner at 2mm isotropic resolution, TR/TE = 7600/88 ms, and 64 gradient directions at $b=1000\;s/{\mathrm{mm}}^{2}$. The IHAD dataset includes 51 adolescent depression patients (age: 14.00±1.41 years; 25 females, 26 males) acquired at University of Electronic Science and Technology of China, with data acquired on a 3T GE scanner at 2mm isotropic resolution, TR/TE = 9635/100 ms, and 64 gradient directions at $b=1000\;s/{\mathrm{mm}}^{2}$.

#### 3.1.2 DMRI Preprocessing and DTI Parameter Computation

For HCP‐YA, we use the provided data that have been processed following the well‐designed HCP minimum processing pipeline [59], including brain masking, motion correction, eddy current correction, EPI distortion correction, and rigid registration to the MNI space [51]. For ABIDE, PPMI, CNP, and IHAD, the dMRI data are processed as described in our previous study using a well‐established pipeline [60] (https://github.com/pnlbwh/pnlpipe), including eddy current‐induced distortion correction, motion correction, and echo‐planar imaging EPI distortion correction. We note that because DDSurfer relies on DTI parameter maps for surface reconstruction, the data preprocessing is essential to correct potential artifacts and distortions to ensure high‐quality DTI model fit.

For each scan, we compute the input DTI parameter maps (FA, MD, and three eigenvalues) in the native dMRI space, which are rigidly registered to the MNI template using Advanced Normalization Tools (ANTs) [61] (except for HCP‐YA that is in the MNI space). These maps are then upsampled to a 1 mm isotropic resolution and normalized using a z‐transform.

### 3.2 Comparison to State‐of‐the‐Art Methods

We compare our DDSurfer method with the following state‐of‐the‐art methods. (1) T1w‐Reg is the most widely used approach to obtain cortical surfaces in dMRI data. It first reconstructs the surfaces from T1w images using FreeSurfer and then nonlinearly registers them to the dMRI space using ANTs. (2) T1w‐Syn represents the synthesis‐based alternative route for generating cortical surfaces in dMRI. In our study, we synthesize a T1w image from dMRI using the recently proposed DeepAnat [22] and then reconstruct cortical surfaces with FreeSurfer [24]. (3) CRUISE [43] is a classic cortical surface reconstruction method from SDFs derived from the MRI data. In our study, we compute SDFs following the pGT surface‐generation pipeline, followed by surface extraction via marching cubes. (4) CoSeg [39], a recently proposed deep learning method that reconstructs cortical surfaces from a segmentation‐defined cortical ribbon using T1w data. In our study, we adapt it to dMRI data by training on the same multi‐channel DTI inputs (FA, MD, and eigenvalues). Furthermore, we include two DDSurfer variants with alternative networks replacing the CMUNeXt 3D backbone. (5) DDSurfer (ConvUNeXt) replaces backbone with 3D ConvUNeXt [62]. (6) DDSurfer (MedNeXt) replaces the backbone with the MedNeXt [63].

Because large‐scale ground‐truth surfaces are unavailable in dMRI data, we quantitatively evaluate each method's results using the following heuristic metrics. First, we compute T1w‐based cortical surfaces using FreeSurfer and measure the WM volume, the pial volume, and the cortical thickness. Using these T1w‐derived measures as reference, we assess each dMRI‐based method by computing the similarity of its outputs to this reference. Specifically, the similarity is defined as $1-\left|M_{T1w}-M_{\mathrm{dMRI}}\right|/\left|M_{T1w}+M_{\mathrm{dMRI}}\right|$, where M is the volume of the WM surface, the volume of the pial surface, or the average cortical thickness across all cortical points. This results in three quantitative metrics including the volume similarity (VS) of WM and pial, and the cortical thickness similarity (CTS), where a higher value indicates a better agreement thus a better result. Second, we compute the distribution of microstructure measures across vertices on cortical regions of interest (ROIs) to evaluate regional homogeneity on the surfaces. For both the WM and pial surfaces, we first subdivide them into standard lobes (including frontal, parietal, temporal, occipital, cingulate, and insula) via post‐surface processing (see Section 2.3). For each ROI, diffusion microstructure measures are assigned to every vertex using those from its corresponding voxel. We then quantify homogeneity of each region using the regional coefficient of variation (rCoV), defined as the interquartile range divided by the median (IQR/Median) of the vertex‐wise diffusion values, as used in [64]. We compute an FA‐based rCoV for the WM surface, and an MD‐based rCoV for the pial surface, where a lower rCoV indicates a higher homogeneity. We also extend the homogeneity analysis from lobar regions to the entire cortical ribbon (overall evaluation). For this, we compute the relative standard deviation (RSD) of each diffusion measure (i.e., FA and MD) across all voxels located between the WM and pial surfaces, as used in [18]. A lower RSD indicates a higher overall homogeneity and is therefore considered a better result.

This evaluation is performed on the HCP‐YA test set. All compared methods use the same input preprocessed dMRI data and the learning‐based methods (DDSurfer, CoSeg, and the two additional DDSurfer variants) are trained with their optimized hyperparameters to ensure fair comparison. For each metric, we report the mean and standard deviation across the testing subjects. To evaluate if there is significant improvement over the compared methods, we perform paired t‐tests comparing our method against the second performing compared method (i.e., T1w‐Reg, CRUISE, or CoSeg), where differences are considered statistically significant when p<0.05.

Table 1 summarizes the quantitative comparison with state‐of‐the‐art methods. In terms of volumetric agreement, DDSurfer achieves the highest similarity to the T1w‐based reference for both WM and pial surfaces, indicating that its reconstructed cortical volumes most closely match those from the T1w‐based reference. For diffusion‐derived measures, DDSurfer consistently yields the lowest rCoV across the cortical lobes, reflecting more homogeneity within‐ribbon FA (WM) and MD (pial) and fewer partial‐volume or misregistration artifacts. In the overall evaluation, DDSurfer also attains the highest cortical thickness similarity and the lowest global RSD_FA_ and RSD_MD_, demonstrating improved stability of morphometric and diffusion‐derived metrics compared with all compared methods.

**TABLE 1:** State‐of‐the‐art method comparison results. For each metric, the best performing method is highlighted in bold, the best‐performing external baseline among the four methods on the left is underlined, and * denotes statistical significance in paired t‐tests between DDSurfer and that underlined baseline (** indicates p≤0.01, * indicates p≤0.05).

**Metric**	**T1w‐Reg**	**T1w‐Syn**	**CRUISE**	**CoSeg**	**DDSurfer (Ours)**		
					ConvUNeXt	MedNeXt	**Proposed**
**WM Surface Evaluation**							
Volume Similarity (VS) ↑	—	0.97 ± 0.01	0.94 ± 0.05	0.96 ± 0.02	0.98 ± 0.01	0.98 ± 0.02	**0.99** ± **0.01** 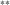
***Local Homogeneity (rCoV on FA)*** ↓							
Cingulate	1.93 ± 0.21	1.78 ± 0.13	1.91 ± 0.29	1.82 ± 0.19	1.71 ± 0.17	1.68 ± 0.16	**1.64** ± **0.15** 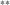
Frontal	1.97 ± 0.18	1.84 ± 0.15	1.93 ± 0.25	1.88 ± 0.19	1.85 ± 0.17	1.82 ± 0.15	**1.78** ± **0.16** 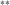
Insula	1.05 ± 0.12	1.05 ± 0.12	1.06 ± 0.16	1.04 ± 0.11	1.03 ± 0.10	1.02 ± 0.09	**1.01** ± **0.08** 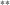
Occipital	0.85 ± 0.09	0.81 ± 0.06	0.85 ± 0.12	0.84 ± 0.08	0.83 ± 0.07	0.81 ± 0.13	**0.79** ± **0.06** 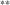
Parietal	1.20 ± 0.14	1.17 ± 0.08	1.18 ± 0.18	1.19 ± 0.11	1.18 ± 0.09	1.17 ± 0.18	**1.16** ± **0.10** 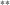
Temporal	1.29 ± 0.13	1.15 ± 0.07	1.24 ± 0.17	1.21 ± 0.08	1.18 ± 0.15	1.15 ± 0.12	**1.11** ± **0.06** 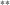
**Pial Surface Evaluation**							
Volume Similarity (VS) ↑	—	0.98 ± 0.01	0.92 ± 0.05	0.96 ± 0.03	0.98 ± 0.01	0.98 ± 0.01	**0.98** ± **0.01** 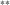
***Local Homogeneity (rCoV on MD)*** ↓							
Cingulate	0.84 ± 0.10	0.85 ± 0.04	0.85 ± 0.13	0.85 ± 0.09	0.84 ± 0.08	0.84 ± 0.08	**0.84** ± **0.07** 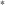
Frontal	1.94 ± 0.62	1.61 ± 0.13	2.05 ± 0.75	1.82 ± 0.51	1.70 ± 0.35	1.64 ± 0.28	**1.57** ± **0.18** 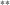
Insula	0.45 ± 0.05	0.39 ± 0.05	0.46 ± 0.08	0.43 ± 0.04	0.41 ± 0.04	0.38 ± 0.03	**0.35** ± **0.03** 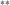
Occipital	0.52 ± 0.06	0.50 ± 0.04	0.54 ± 0.09	0.51 ± 0.05	0.50 ± 0.05	0.49 ± 0.04	**0.48** ± **0.04** 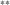
Parietal	0.46 ± 0.05	0.45 ± 0.04	0.48 ± 0.08	0.45 ± 0.05	0.44 ± 0.04	0.43 ± 0.04	**0.42** ± **0.04** 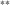
Temporal	1.72 ± 0.35	1.38 ± 0.17	1.75 ± 0.48	1.58 ± 0.29	1.45 ± 0.21	1.36 ± 0.17	**1.29** ± **0.15** 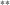
**Overall Evaluation**							
Cortical Thickness Similarity (CTS) ↑	—	0.95 ± 0.02	0.87 ± 0.04	0.93 ± 0.02	0.95 ± 0.02	0.96 ± 0.02	**0.96** ± **0.02** 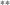
${\mathrm{RSD}}_{\mathrm{FA}}$ ↓	0.44 ± 0.08	0.43 ± 0.06	0.55 ± 0.09	0.43 ± 0.07	0.43 ± 0.05	0.41 ± 0.07	**0.41** ± **0.05** 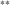
${\mathrm{RSD}}_{\mathrm{MD}}$ ↓	0.25 ± 0.07	0.23 ± 0.04	0.32 ± 0.09	0.24 ± 0.06	0.23 ± 0.03	0.23 ± 0.05	**0.22** ± **0.05** 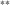

**FIGURE 4:**
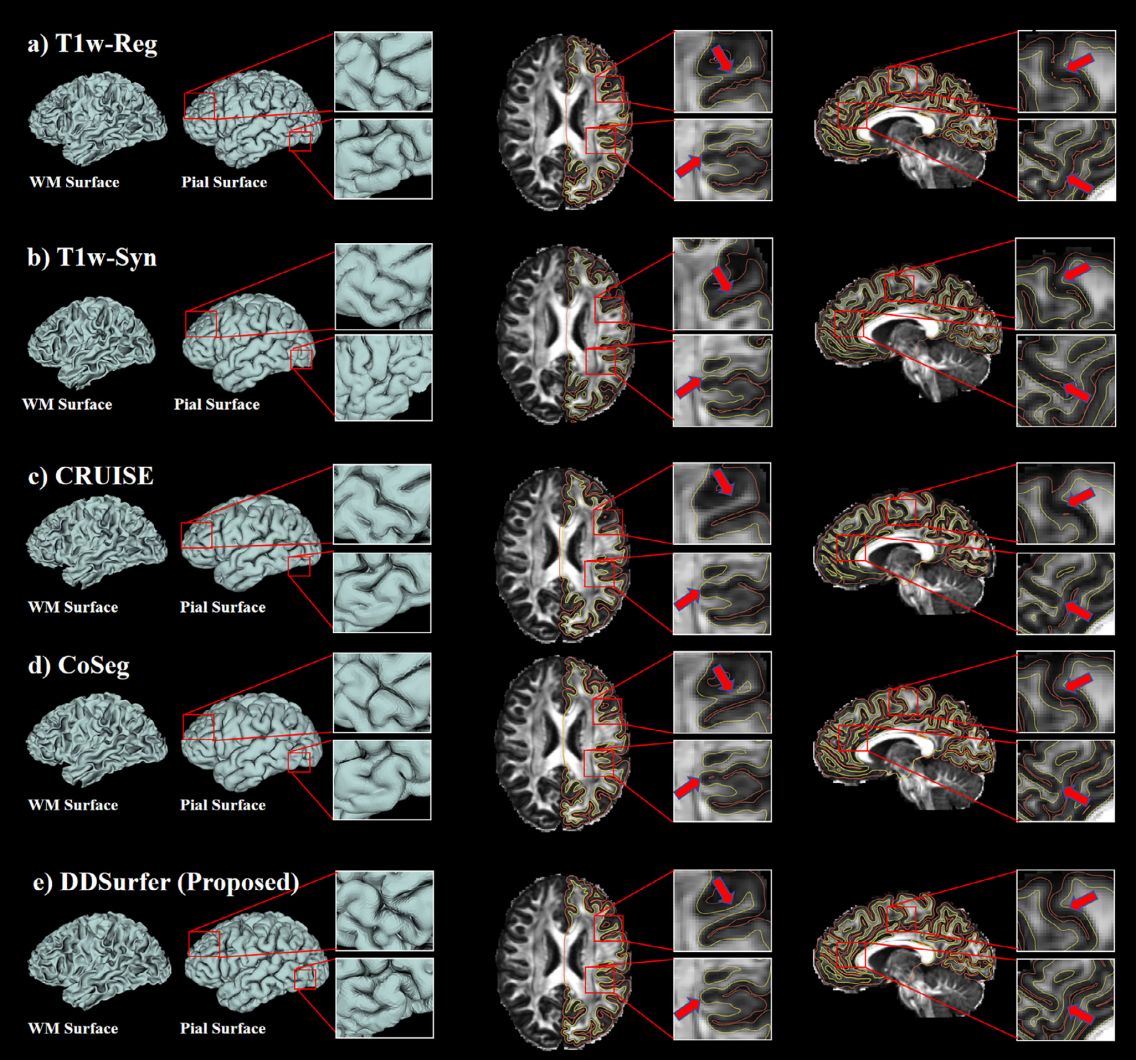
Visual comparison with the state‐of‐the‐art methods on example HCP‐YA data. The figure compares 3D surface renderings and slice overlays on FA maps for (a) T1w‐Reg, (b) T1w‐Syn, (c) CRUISE, (d) CoSeg (dMRI‐adapted), and (e) our DDSurfer. Red arrows highlight example regions where DDSurfer demonstrates superior alignment with the underlying anatomy.

Figure 4 provides a visual comparison on an example HCP‐YA dataset, showing 3D surface renderings and FA‐slice overlays for each method. Although T1w‐Reg benefits from high‐quality T1w anatomy, local misalignments between the warped surfaces and the dMRI can still be observed, particularly in regions with complex folding. The T1w‐Syn route provides a synthesis‐based structural comparison and avoids direct T1w‐to‐dMRI warping, but the highlighted regions still show visible boundary deviations from the underlying dMRI anatomy. CRUISE and CoSeg often exhibit jagged or spatially inconsistent WM and pial boundaries, especially near deep sulci and thin cortical ribbons. In contrast, DDSurfer produces smooth, well‐aligned WM and pial surfaces that follow the dMRI‐derived anatomy more closely. The zoomed insets highlight example regions near the deep sulci, where DDSurfer adheres more closely to WM–GM boundaries and reduces spurious incursions into the CSF.

### 3.3 Ablation Studies

We conduct a series of ablation comparisons to evaluate the contribution of each major design component in DDSurfer. The compared methods include: (1) the pGT pipeline as reference, which applies CRUISE and marching cubes to reconstruct the surfaces using the dMRI‐derived tissue probability maps (as introduced in Section 2.1); (2) a single‐stream baseline model that replaces the dual‐stream encoder to assess the benefit of disentangled anisotropy and diffusivity feature processing; (3) a model without the Cross‐SE Fusion Module, where the two feature streams are directly concatenated, to evaluate the importance of cross‐modality channel interaction; (4) a model without the Large Kernel Attention (LKA) block in the bottleneck, to examine the influence of global context modeling; (5) an FA‐only input model to investigate the complementary role of diffusivity‐derived features; and (6) a SynthSeg‐based variant that keeps the same DDSurfer architecture but replaces the proposed pGT surfaces with those computed based on the SynthSeg method [65]. In this variant, voxelwise probability maps are obtained by softmax fitting of the SynthSeg outputs and then passed through the same downstream SDF‐based pGT generation pipeline.

This evaluation is performed on the HCP‐YA test set. The quantitative metrics of VS and rCoV of WM and pial surfaces, CTS and RSD of FA and MD in the cortical ribbon (as introduced in Section 3.2) are used. For each metric, we report the mean and standard deviation across the testing subjects.

The quantitative results in Table 2 indicate that each component contributes meaningfully to surface reconstruction performance. The pGT surfaces exhibit the worst performance, indicating a critical need for improvement in this initial generation process. Compared with the full DDSurfer, the Single‐stream implementation and the implementation w/o Cross‐SE variants show higher mean rCoV and worse CTS/RSD, highlighting the benefits of disentangled dual‐stream encoding and cross‐SE fusion. Removing D‐LKA leads to moderate but consistent degradation across metrics, whereas the FA‐only model shows the largest drop, especially in rCoV and RSD. Replacing the proposed pGT process with a SynthSeg‐based variant leads to a degradation in surface reconstruction performance, but the changes are not as large as the other components.

**TABLE 2:** Ablation comparison results.

		Metrics	
Surface	Method	VS ↑	Mean rCoV ↓
**WM**	pGT	0.944 ± 0.052	1.362 ± 0.195
	Single‐stream	0.978 ± 0.011	1.425 ± 0.162
	w/o Cross‐SE	0.981 ± 0.010	1.352 ± 0.145
	w/o D‐LKA	0.983 ± 0.009	1.310 ± 0.138
	FA‐only Input	0.971 ± 0.012	1.480 ± 0.175
	SynthSeg‐based	0.983 ± 0.011	1.263 ± 0.093
	**DDSurfer (Proposed)**	**0.985** ± **0.008**	**1.248** ± **0.113**
**Pial**	pGT	0.923 ± 0.051	1.022 ± 0.268
	Single‐stream	0.975 ± 0.011	1.150 ± 0.135
	w/o Cross‐SE	0.978 ± 0.010	1.025 ± 0.122
	w/o D‐LKA	0.979 ± 0.010	0.958 ± 0.115
	FA‐only Input	0.970 ± 0.012	1.210 ± 0.148
	SynthSeg‐based	0.977 ± 0.008	0.837 ± 0.075
	**DDSurfer (Proposed)**	**0.980** ± **0.011**	**0.825** ± **0.092**
	**Method**	**CTS** ↑	**RSD_FA_** ↓	**RSD_MD_** ↓
**Overall**	pGT	0.872 ± 0.038	0.550 ± 0.087	0.322 ± 0.092
	Single‐stream	0.981 ± 0.011	0.440 ± 0.063	0.290 ± 0.061
	w/o Cross‐SE	0.983 ± 0.010	0.460 ± 0.062	0.270 ± 0.058
	w/o D‐LKA	0.985 ± 0.009	0.450 ± 0.055	0.250 ± 0.053
	FA‐only Input	0.972 ± 0.012	0.530 ± 0.074	0.310 ± 0.071
	SynthSeg‐based	0.983 ± 0.009	0.424 ± 0.052	0.228 ± 0.035
	**DDSurfer (Proposed)**	**0.986** ± **0.008**	**0.410** ± **0.047**	**0.220** ± **0.046**

### 3.4 Test–Retest Assessment

This experiment evaluates the reproducibility (reliability) of DDSurfer using the HCP‐YA test–retest dataset. In dMRI, test–retest reproducibility serves as a critical indicator of methodological reliability, reflecting the consistency of a method's outputs across repeated scans of the same subject [66, 67, 68]. In our study, DDSurfer is applied separately to the test and retest dMRI scans per subject. The resulting cortical surfaces are then spatially co‐registered using their corresponding b0 (non‐diffusion‐weighted) images via a nonlinear transformation in ANTs. For baseline comparison, we include T1w‐Reg and T1w‐Syn, and align the resulting cortical surfaces using the same co‐registration process.

We compute the following quantitative metrics for each subject and each method. First, for both WM and pial surfaces, vertex‐level differences are measured using the Chamfer distance between the corresponding surfaces between test and retest scans. Second, to assess morphological features derived from the reconstructed surfaces, we measure the curvature difference between the scans. Finally, we measure the differences in cortical thickness computed from the two surfaces to assess the overall performance. The differences of the entire cortex and each cortical parcel derived from the DK atlas are computed. For each metric, we report the mean and standard deviation across subjects, and compare DDSurfer with the two structural baselines using two‐sided paired t‐tests, with statistical significance defined at p<0.05.

**FIGURE 5:**
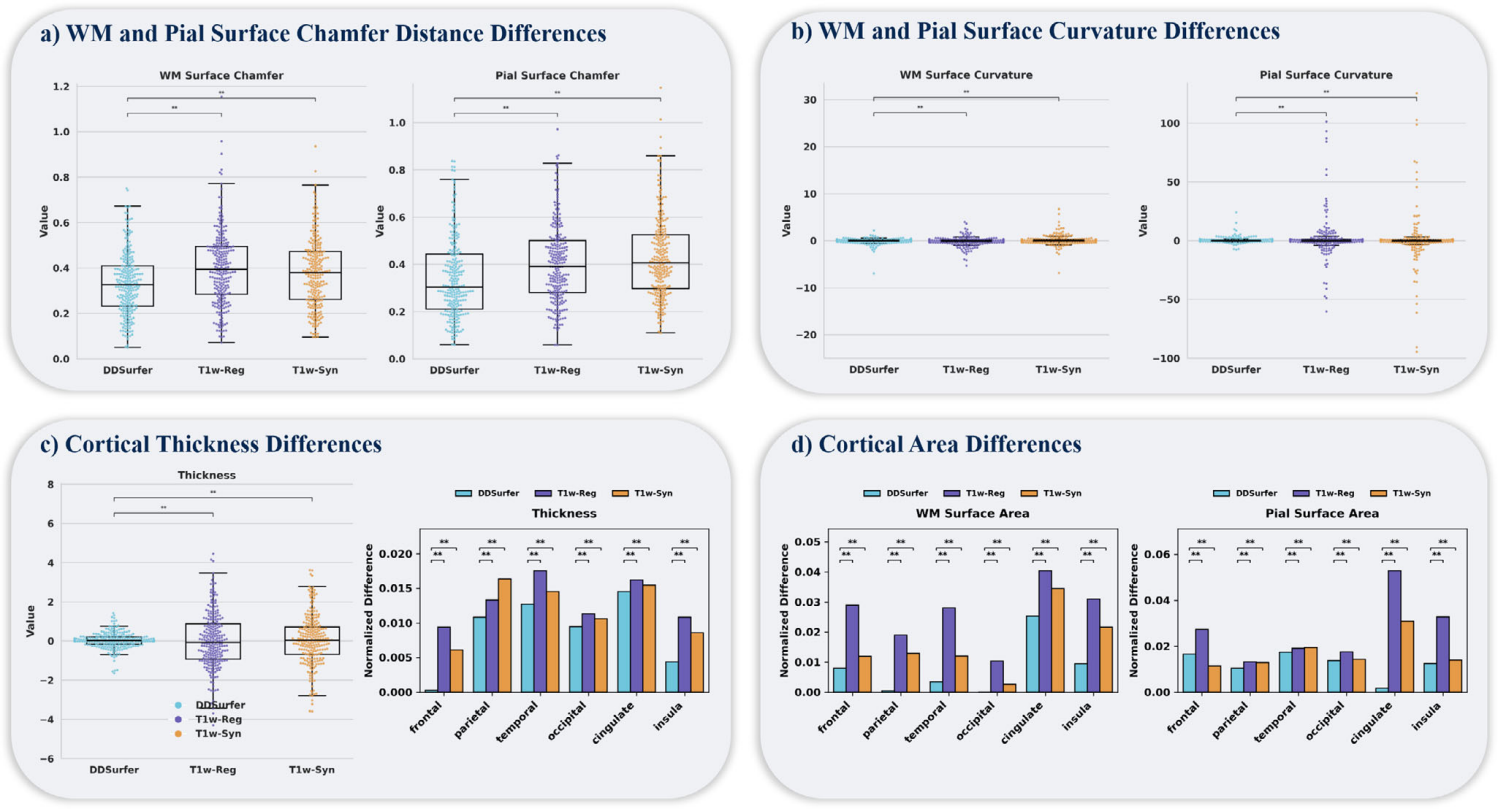
Test–retest comparison of DDSurfer, the registration‐based structural baseline T1w‐Reg, and the synthesis‐based structural baseline T1w‐Syn. The significance brackets denote paired t‐tests between DDSurfer and each structural baseline (** indicates p≤0.01, * indicates p≤0.05).

Figure 5 compares the test–retest reliability of DDSurfer against T1w‐Reg and T1w‐Syn. Figure 5a shows that DDSurfer yields lower vertex‐wise Chamfer distances between test and retest WM and pial surfaces than the structural baselines, with the significance brackets summarizing paired comparisons against T1w‐Reg and T1w‐Syn. In Figure 5b, vertex‐wise differences in mean curvature (Retest – Test) for DDSurfer remain more tightly centered around zero with smaller spread, especially relative to T1w‐Reg. Figure 5c presents vertex‐wise cortical thickness differences, where DDSurfer again exhibits a narrower distribution and smaller bias than the structural baselines, indicating higher test–retest stability. At the regional level (Figure 5d), DDSurfer generally shows smaller percentage differences in cortical thickness and WM/pial surface area across lobes, supporting improved reproducibility of morphometric measures in the three‐method comparison.

### 3.5 Validation Against Expert‐Annotated Surfaces

To complement the indirect morphometric and diffusion‐based evaluations above, we further perform direct geometric validation against expert‐annotated reference surfaces. In collaboration with a neuroanatomist (J.R.), we manually delineate WM and pial surfaces in dMRI space on one example HCP subject using the Segment Editor module in 3D Slicer [69, 70], with reference to the FA and MD images to ensure anatomically consistent boundary placement.

We evaluate DDSurfer and two baseline methods (T1w‐Reg and T1w‐Syn) using three complementary geometric metrics with respect to the manual ground truth. ASSD (average symmetric surface distance) measures mean bidirectional surface agreement, HD90 (90th‐percentile Hausdorff distance) captures large local errors while remaining less sensitive to extreme outliers, and Chamfer RMS (root‐mean‐square surface distance) reflects overall distance magnitude with higher sensitivity to large local deviations. Furthermore, to examine the reconstructed surfaces at each vertex, we plot the symmetric distance per vertex to show the distribution of errors for each compared method. These vertex‐wise distances are also rendered on the reconstructed surfaces, allowing visualization of how errors are spatially distributed over the reconstructed white matter and pial surfaces.

**FIGURE 6:**
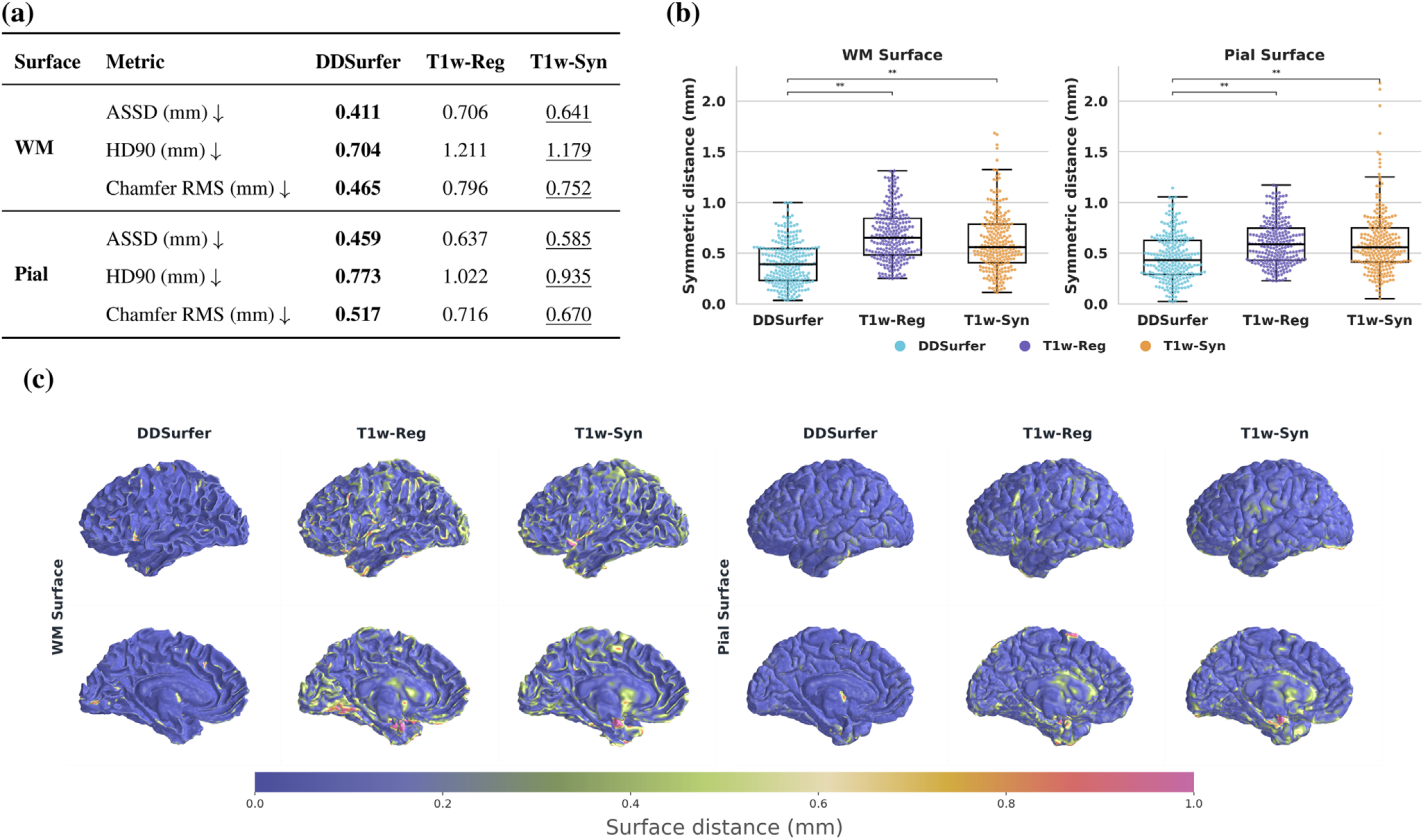
Direct geometric evaluation against expert‐annotated reference surfaces. (a) Quantitative metrics for WM and pial surfaces. (b) Vertex‐wise surface distance distributions. (c) Surface error map comparison for WM and pial surfaces. Significance brackets denote paired t‐tests (** p≤0.01).

Figure 6a shows that DDSurfer achieves the lowest ASSD, HD90, and Chamfer RMS for both WM and pial surfaces. The vertex‐wise distance distributions in Figure 6b show a narrower range for DDSurfer than for T1w‐Reg and T1w‐Syn, indicating consistently smaller geometric errors. This pattern is further supported by the surface error map visualization in Figure 6c, where DDSurfer shows less spatially extensive high‐error regions and overall better agreement with the expert‐annotated reference surfaces. Significance brackets denote paired comparisons of vertex‐wise distance values (** p≤0.01).

### 3.6 Computational Efficiency

A key advantage of DDSurfer is its superior computational efficiency, making it highly practical for large‐scale studies. Table 3 summarizes the approximate inference runtime for DDSurfer and the comparison methods. The mean inference time across the testing HCP‐YA subjects is reported. DDSurfer performs cortical surface reconstruction in approximately 5 s, slightly behind the adapted dMRI‐based CoSeg method using about 4 s. Both are dramatically faster than the baseline T1w‐Reg and T1w‐Syn approaches (approximately 4–6 h) and the CRUISE algorithm (5–6 min).

**TABLE 3:** Inference Runtime Comparison. The computation is performed on a Linux server equipped with RTX 3090 GPUs.

Method	Runtime
T1w‐Reg + FreeSurfer	∼4–6 H
T1w‐Syn + FreeSurfer	∼4–6 H
CRUISE	∼5–6 Min
CoSeg (dMRI‐adapted)	∼4 S
DDSurfer (Proposed)	∼5 S

### 3.7 Generalization Capability

This experiment evaluates DDSurfer's ability to generalize across diverse realistic conditions, including multiple clinical cohorts, acquisition protocols, and scanner types, using data from ABIDE, PPMI, CNP, and IHAD (see Section 3.1). We use T1w‐Reg as the registration‐based structural baseline, where cortical surfaces are computed from T1w and aligned to dMRI via co‐registration using ANTs, and we further include T1w‐Syn (DeepAnat)+FreeSurfer as a synthesis‐based structural baseline under the same cross‐dataset comparison logic.

Given the fact that there are no ground‐truth surfaces in these datasets, we evaluate performance using the heuristic metrics of regional surface homogeneity (see Section 3.2 for metric details), including: (1) rCoV, which measures vertex‐wise homogeneity of diffusion measures on the reconstructed WM and pial surfaces, and (2) RSD, which quantifies voxel‐wise homogeneity of diffusion measures within the cortical ribbon between the WM and pial surfaces. As in Section 3.2, these metrics provide indirect rather than direct geometric evidence because native‐dMRI‐space reference surfaces are unavailable for these cohorts. A lower value of these metrics indicates a higher regional homogeneity thus a better result. The quantitative comparison in this subsection is carried out across DDSurfer, T1w‐Reg, and T1w‐Syn. To evaluate statistical significance, we perform paired t‐tests comparing our method against the compared methods per evaluation metric, where differences are considered statistically significant when p<0.05.

**FIGURE 7:**
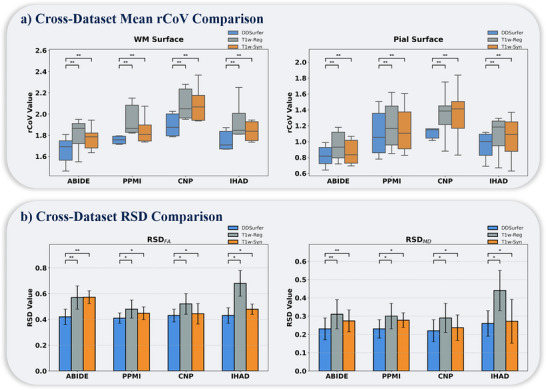
Cross‐dataset generalization performance across multiple testing datasets from different sources. * denotes statistical significance in paired t‐tests between DDSurfer and each baseline method (T1w‐Reg or T1w‐Syn) (** indicates p≤0.01, * indicates p≤0.05).

**FIGURE 8:**
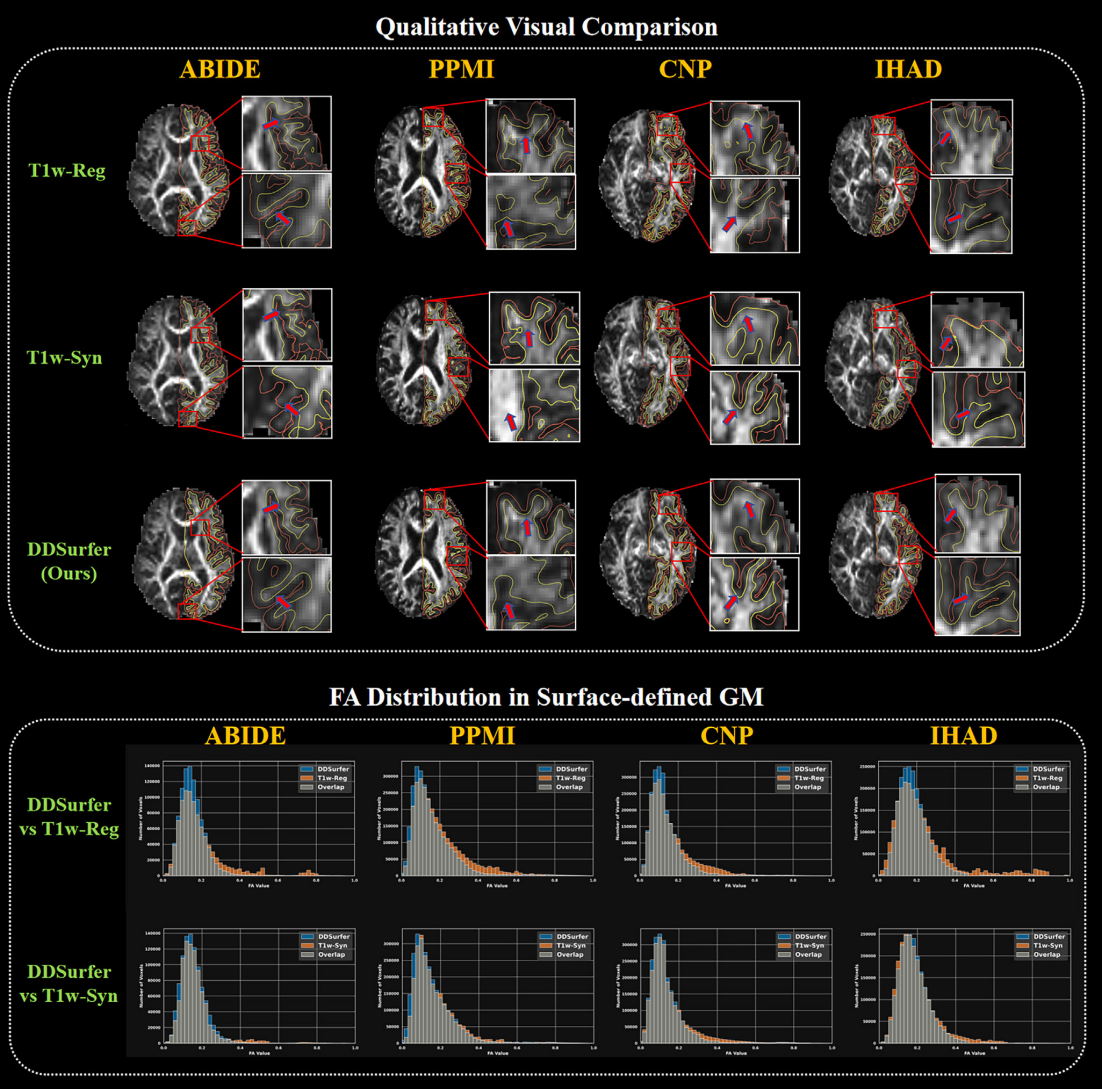
Visualization of cortical surface reconstruction results in a randomly selected case for each data source. The upper panel shows the reconstructed surfaces overlaid on the FA map for the T1w‐Reg, T1w‐Syn and our DDSurfer methods. The bottom panel shows the voxel‐wise FA distribution of the cortical ribbon between the WM and pial surfaces.

Figure 7 provides the evaluation results of DDSurfer, T1w‐Reg, and T1w‐Syn on data from multiple sources, where panel (a) reports rCoV of the WM and pial surfaces, and panel (b) reports RSD of FA and MD of the cortical ribbon region. We can see that DDSurfer significantly outperforms the baseline methods in both vertex‐ and voxel‐wise metrics across all datasets. Figure 8 gives a visualization of the results from each method, which represent the quantitative trends, with qualitative overlays in the upper panel and the corresponding FA histograms in the lower panel. The T1w‐Reg method frequently misaligns with FA boundaries, and the T1w‐Syn method also shows misalignment in the highlighted regions, though it is visually better than T1w‐Reg; by contrast, DDSurfer adheres to FA transitions, preserves thin gyral/sulcal structures, and avoids spurious bridges. The improvement of DDSurfer over the compared baselines is more visually apparent in the deep sulci regions, as highlighted in the zoomed insets. The accompanying FA histograms (bottom row) show tighter, less skewed GM distributions under DDSurfer in both the DDSurfer vs. T1w‐Reg and DDSurfer vs. T1w‐Syn comparisons. Together, these results confirm strong cross‐dataset generalization and stable surface reconstruction quality.

### 3.8 Downstream Application Assessment

We further evaluate the impact of DDSurfer surfaces on surface‐based tractography, a downstream application that is highly sensitive to the accuracy of anatomical constraints. Surface‐based tractography leverages cortical surface meshes to define anatomically meaningful seeding and termination at the gray–white interface, which is particularly beneficial for reconstructing superficial/short association fibers close to the cortical surface [3]. Traditionally, this surface prior is obtained by mapping FreeSurfer T1w‐derived surfaces into diffusion space via T1‐to‐dMRI registration (i.e., the T1w‐Reg method). In our setting, we integrate DDSurfer by replacing the T1w‐Reg surfaces with DDSurfer‐predicted WM and pial surfaces in diffusion space, and use them for surface seeding/termination and the same surface‐informed anatomical constraints, while keeping the rest of the tractography pipeline unchanged. For comparison, we also performed the tractography based on the T1w‐Reg‐based surfaces as used in the official implementation [3].

The evaluation is performed on the HCP‐YA test set. The compared methods use the same preprocessed dMRI data and identical tractography parameters to ensure a fair comparison; the only difference lies in the source of the anatomical surfaces (T1w‐Reg vs. DDSurfer). For each subject, we generate 10 million streamlines and compute metrics at multiple sampling levels (streamline counts). The evaluated surface‐based metrics include: (i) coverage (%), defined as the proportion of white surface vertices hit by at least one streamline endpoint, where a higher value means a better result; (ii) mean distance to the nearest white surface vertex (mm), where a lower distance means a better result; (iii) coverage bias, defined as the gyri‐coverage ratio normalized by the global gyri ratio (gyri: curvature<0), where a lower value means a better result; and (iv) streamline ends in gyri (%), defined as the fraction of all endpoints assigned to gyri, where a higher value means a better result.

**FIGURE 9:**
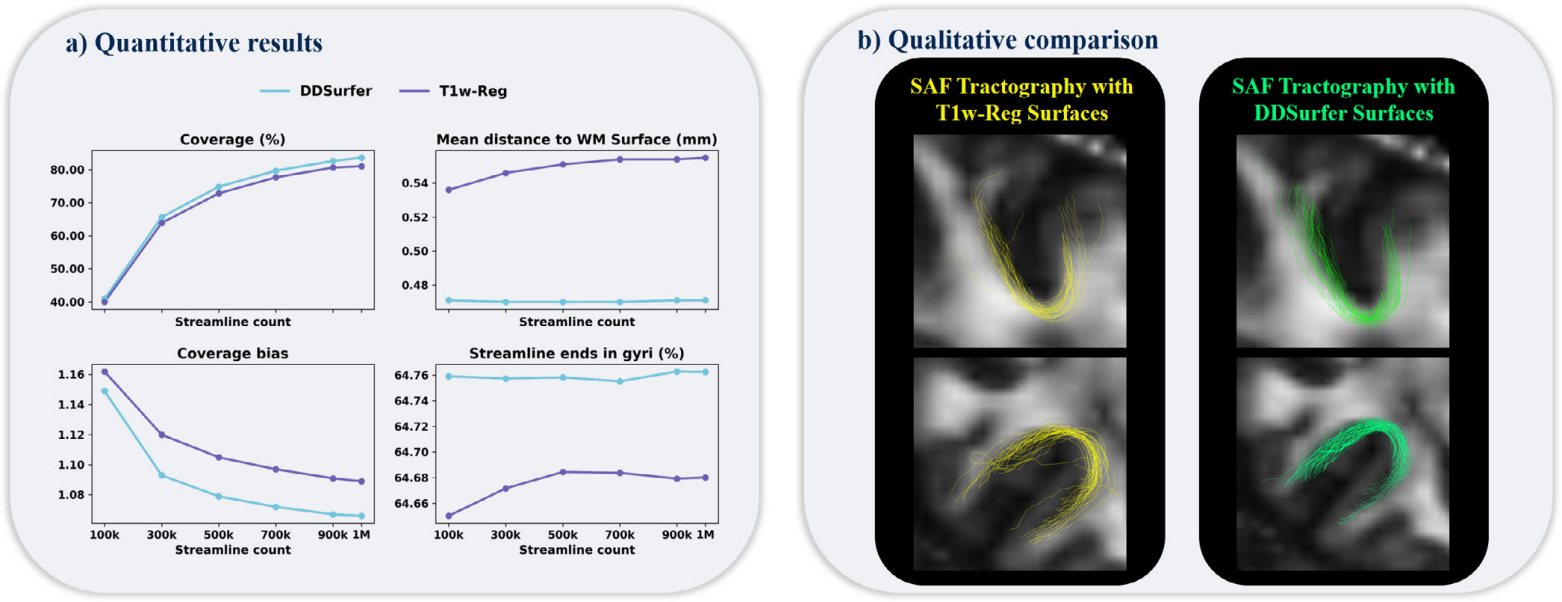
Surface‐based tractography evaluation of the T1w‐Reg and DDSurfer methods. (a) shows the quantitative comparison results, and (b) gives a visualization of superficial U‐fibers identified using the two methods.

Figure 9a shows that DDSurfer consistently outperforms the T1w‐Reg baseline across the evaluated tractography metrics. Figure 9b provides qualitative evidence that complements the quantitative findings. Under identical tractography settings, DDSurfer produces more coherent and anatomically plausible streamline trajectories with fewer irregular terminations than T1w‐Reg. Collectively, these results suggest that the more accurate and intrinsically aligned anatomical priors provided by DDSurfer enable a more complete and biologically plausible connectome, thereby improving the reliability of tractography‐based analyses.

## 4 Discussion

In this work, we introduce DDSurfer, a novel deep learning framework for cortical surface reconstruction directly from dMRI data. DDSurfer eliminates the inter‐modality registration required by conventional methods, mitigating associated errors from dMRI distortion and low resolution. Furthermore, DDSurfer enables surface reconstruction directly from dMRI when anatomical MRI is not available. Our experimental results demonstrate that DDSurfer achieves excellent surface reconstruction performance, significantly outperforming conventional pipelines and other currently available deep learning approaches. Furthermore, DDSurfer exhibits robust generalization to diverse clinical datasets and shows superior test–retest reliability. The high computational efficiency and validated utility in downstream applications, including surface‐based tractography, underscore its practical value for neuroimaging research.

DDSurfer is a weakly‐supervised framework that learns surface reconstruction in dMRI without ground‐truth surfaces. We use a weakly‐supervised framework because there are currently no tools that extract accurate surfaces directly from dMRI data. This is unlike deep‐learning methods developed to generate surfaces from T1w data, that can use tools like FreeSurfer to generate high‐quality anatomical surfaces to guide supervised learning. Our approach is to generate imprecise surfaces to approximate cortical boundaries followed by a weakly‐supervised learning strategy that refines these initial estimates. This strategy thus uses the imperfect pGT surfaces as soft geometric targets while effectively deforming the WM and pial surfaces in challenging regions such as deep cortical sulci and narrowly spaced gyri. As a result, this lightweight regularization scheme achieves robustness to noisy pGT, while preserving plausible topological integrity. Our experiments show that the weakly‐supervised learning framework generalizes well to different methods for generating pGT, provided they yield reasonably good pGT overall. However, higher‐quality pGT will lead to better surface reconstruction outcomes.

We show that our DDSurfer method outperforms the compared methods due to several novel network designs. First, compared with the original CMUNeXt network [44], DDSurfer incorporates a DualStream–FuseNet subnetwork that jointly encodes dMRI anisotropy and diffusivity information. This joint feature learning enhances the differentiation between tissue types, improving the delineation of surface boundaries. In the literature, leveraging complementary information from multiple derived dMRI parameters has been shown to enhance computational tasks such as segmentation [18], registration [71], and disease classification [72]. Our DualStream‐FuseNet jointly encodes anisotropy and diffusivity cues and fuses them with lightweight cross‐stream gating at multiple scales, allowing complementary boundary information to reinforce each other without heavy computation. Furthermore, DDSurfer includes a hierarchical network design to leverage the multi‐scale features for coarse‐to‐fine deformation estimation. Compared with prior single‐stream or single‐scale designs [39], we fuse features across streams and resolutions and aggregate multi‐scale decoder outputs for coarse‐to‐fine deformation. In our experiments, we propose several heuristic metrics to evaluate the generated surfaces. The quantitative comparisons show consistent gains over baselines across morphology similarity and diffusion homogeneity metrics, indicating improved boundary placement. Further, qualitative results corroborate reduced misalignment and oversmoothing. This advantage is particularly visible in deep sulci: the zoomed examples in Figures 4 and 8 both show that DDSurfer follows these difficult sulcal boundaries more faithfully than T1w‐Reg and T1w‐Syn, which remain more prone to local deviation or oversmoothing in these regions.

We demonstrate that DDSurfer generalizes effectively to diverse dMRI datasets, spanning different subject populations, age ranges, and acquisition protocols across multiple scanners. This capability is critical for clinical and research applications, as dMRI lacks a universal acquisition standard; parameters like pulse timing, diffusion gradient strength, and spatial resolution vary widely, posing a significant challenge to learning‐based methods. Furthermore, we show that DDSurfer achieves largely improved cortical surface estimation on dMRI data with different spatial resolutions. The low spatial resolution of dMRI poses inherent challenges for cortical surface reconstruction. One important consequence is partial volume effects, where voxels near the GM and WM boundaries contain mixed tissue signals to bias tissue boundary estimates. As a result, reconstructed surfaces may overestimate or underestimate thickness in some areas. Multiple efforts have been made to mitigate it, e.g., using fuzzy or probabilistic tissue representations combined with topology‐preserving surface evolution [43, 73], template‐based deformation models [39], and boundary‐aware constraints in deep sulci [39]. DDSurfer follows the similar general rationale: a template‐based diffeomorphic deformation enforces topological constraints for the WM surface, while a one‐way boundary loss and inflation prior help reduce pial surface underestimation in deep sulci. As a result, DDSurfer obtains the closest cortical thickness agreement with those derived from high‐resolution T1w data, under both high‐quality HCP (1.25 ${\mathrm{mm}}^{3}$ resolution) and the multiple clinical acquisition datasets (ABIDE, CNP, PPMI and IHAD; 2 ${\mathrm{mm}}^{3}$ resolution). Because DDSurfer predicts surfaces based on the dMRI‐derived parameter maps, its local boundary precision can still be affected when there are residual motion or distortion artifacts. Nevertheless, our evaluation across multiple datasets with different acquisition protocols suggests that, with standard dMRI preprocessing, DDSurfer is generally applicable across heterogeneous data. Our approach achieves robust generalizability through several key design choices. First, it is trained on the high‐quality, multi‐shell HCP‐YA dataset, a strategy proven effective for generalizing dMRI learning tasks such as tissue segmentation [18, 20] and tract parcellation [74, 75]. Second, by building upon the conventional DTI model—chosen for its simplicity and compatibility—our framework maintains applicability to a broad spectrum of data, including legacy acquisitions.

The DDSurfer outputs are FreeSurfer‐compatible WM and pial surface meshes, which can be directly imported into standard FreeSurfer post‐processing. This enables a broad set of established surface‐based analyses, including atlas‐based parcellation and regional statistics, as well as morphometric measurements such as cortical thickness, curvature, and surface area, using off‐the‐shelf FreeSurfer utilities. In addition, the diffusion‐native surfaces provide anatomically grounded priors for surface‐based tractography (e.g., surface seeding/termination and surface‐informed constraints), facilitating downstream connectomics analyses without relying on potentially unreliable T1w‐to‐dMRI registration. Overall, this compatibility makes DDSurfer a practical drop‐in component for diffusion‐centric morphometry and connectome studies.

DDSurfer offers an easily accessible and computationally efficient tool, enabling large‐scale dMRI analysis. Our method is able to perform cortical surface reconstruction in seconds, drastically reducing the processing time from the several hours required by the conventional T1w‐Reg pipeline, which relies on FreeSurfer and inter‐modality registration. Furthermore, our trained model has been integrated into 3D Slicer as the SlicerDDSurfer module, providing an interactive GUI workflow from preprocessing to surface export.

Potential limitations of the present study, including suggested future work to address limitations, are as follows. First, the current input is based on the DTI model; future work could explore incorporating features from more advanced multi‐compartment models to potentially capture even finer microstructural details. Second, the model's performance on brains with significant pathology, such as large lesions or tumors, is not evaluated and remains an important direction for clinical translation. Third, because the low spatial resolution of dMRI poses an inherent challenge for cortical surface reconstruction, an interesting future direction is to improve acquisition protocols to obtain high‐resolution dMRI data or to combine our method with super‐resolution techniques, thereby enabling improved dMRI‐based cortical surface reconstruction. Finally, while the framework is tested on a range of ages, its performance on very young (e.g., neonatal) or very old populations may require further validation and potential fine‐tuning.

## 5 Conclusion

In conclusion, DDSurfer provides a powerful, accurate, and efficient solution for direct dMRI‐based cortical surface reconstruction. By overcoming the fundamental limitations of previous methods, it facilitates more robust and accessible surface‐based analyses, with significant potential to advance connectomics, morphometry, and multimodal studies centered on the rich information inherent in dMRI data.

## Conflicts of Interest

The authors declare no conflicts of interest.

## Supporting information




**Supporting File**: advs76596‐sup‐0001‐SuppMat.pdf.

## Data Availability

The data that support the findings of this study are available on request from the corresponding author. The data are not publicly available due to privacy or ethical restrictions.
