# Supplementary material for: DDSurfer: A Weakly‐Supervised Dual‐Stream Deep Learning Framework for Cortical Surface Reconstruction From Diffusion MRI
Source: Adv Sci (Weinh). 2026 Jul 23:e76596. Online ahead of print. doi: 10.1002/advs.76596 (PMC13393330; doi:10.1002/advs.76596)
Supplement: Supplementary file 1 — Supporting File: advs76596‐sup‐0001‐SuppMat.pdf. [file ADVS-9999-e76596-s001.pdf]

Supplementary Materials

Assessment of the impact of pseudo-ground-truth surfaces on weak supervision

---

Supporting information for

DDSurfer: A Weakly-Supervised Dual-Stream Deep Learning Framework for Cortical Surface Reconstruction from Diffusion MRI

Chengjin Li[1], Wei Zhang[1], Xi Zhu[1], Yuqian Chen[2], Nir A. Sochen[3], Jarrett Rushmore[2,4], Carl-Fredrik Westin[2], Yogesh Rathi[2], Lauren J. O'Donnell[2], Ofer Pasternak[2], and Fan Zhang[1]

[1]University of Electronic Science and Technology of China, Chengdu, China
[2]Brigham and Women's Hospital, Harvard Medical School, Boston, MA, USA
[3]School of Mathematical Sciences, University of Tel Aviv, Tel Aviv, Israel
[4]Department of Anatomy and Neurobiology, Boston University School of Medicine, Boston, MA, USA

In the controlled label-noise sensitivity experiment, the training pseudo-ground-truth surface vertices were perturbed along their local surface normal directions using scalar Gaussian displacement noise. For each vertex $\mathbf{v}_i$ with unit normal vector $\mathbf{n}_i$, the perturbed vertex $\widetilde{\mathbf{v}}_i$ was defined as

$$\widetilde{\mathbf{v}}_i = \mathbf{v}_i + \epsilon_i \mathbf{n}_i, \quad \epsilon_i \sim \mathcal{N}(0, \sigma^2), \tag{1}$$

where σ was set to 0, 0.5, 1.0, and 2.0 mm. The face topology was kept unchanged, and only the vertex coordinates of the training pseudo-labels were perturbed. The input diffusion-derived features, validation data, and evaluation labels were kept unchanged, so that the observed performance changes primarily reflected the effect of noisy training supervision.

As shown in Supplementary Figure S1, mild boundary perturbations of 0.5–1.0 mm resulted in only minor changes in diffusion-ribbon RSD, whereas the 2.0 mm perturbation led to a more evident increase. These results suggest that DDSurfer has a degree of robustness to mild boundary-level label inaccuracies, while severe pseudo-label corruption can still degrade reconstruction quality.

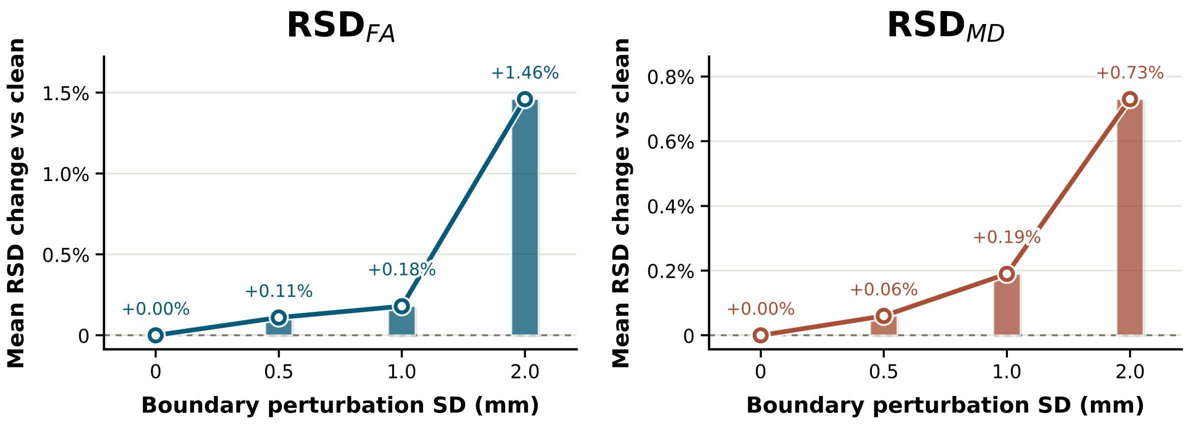

Figure S1. Label-noise sensitivity analysis based on diffusion-ribbon RSD.

In the iterative self-training experiment, after the initial DDSurfer training, the reconstructed

surfaces were used as updated pseudo-labels for two additional rounds of training, with 20 extra epochs in each round. The input diffusion-derived features and validation labels were kept unchanged, so that the comparison focused on the effect of iterative pseudo-label refinement.

As shown in Supplementary Figure S2, diffusion-ribbon RSD showed only small changes across the direct inference stage and the two self-training rounds. This result is consistent with the label-noise sensitivity experiment above, where small perturbations to high-quality pseudo-ground-truth surfaces have only limited impacts (≤0.2%) on reconstruction performance. Since the DDSurfer-derived surfaces after the initial training are already of high quality, using them as updated pseudo-labels introduces only limited additional supervisory information.

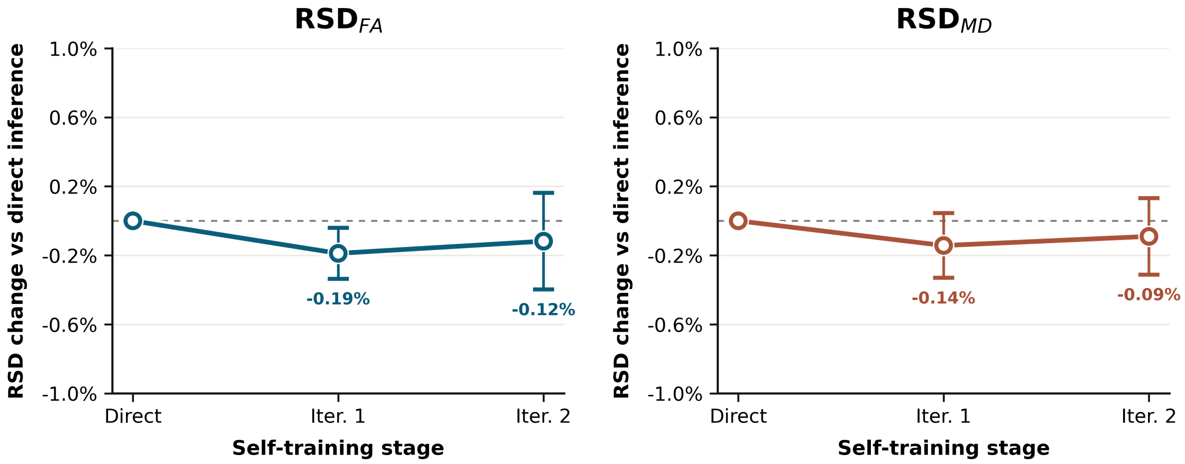

Figure S2. Iterative self-training analysis based on diffusion-ribbon RSD.
